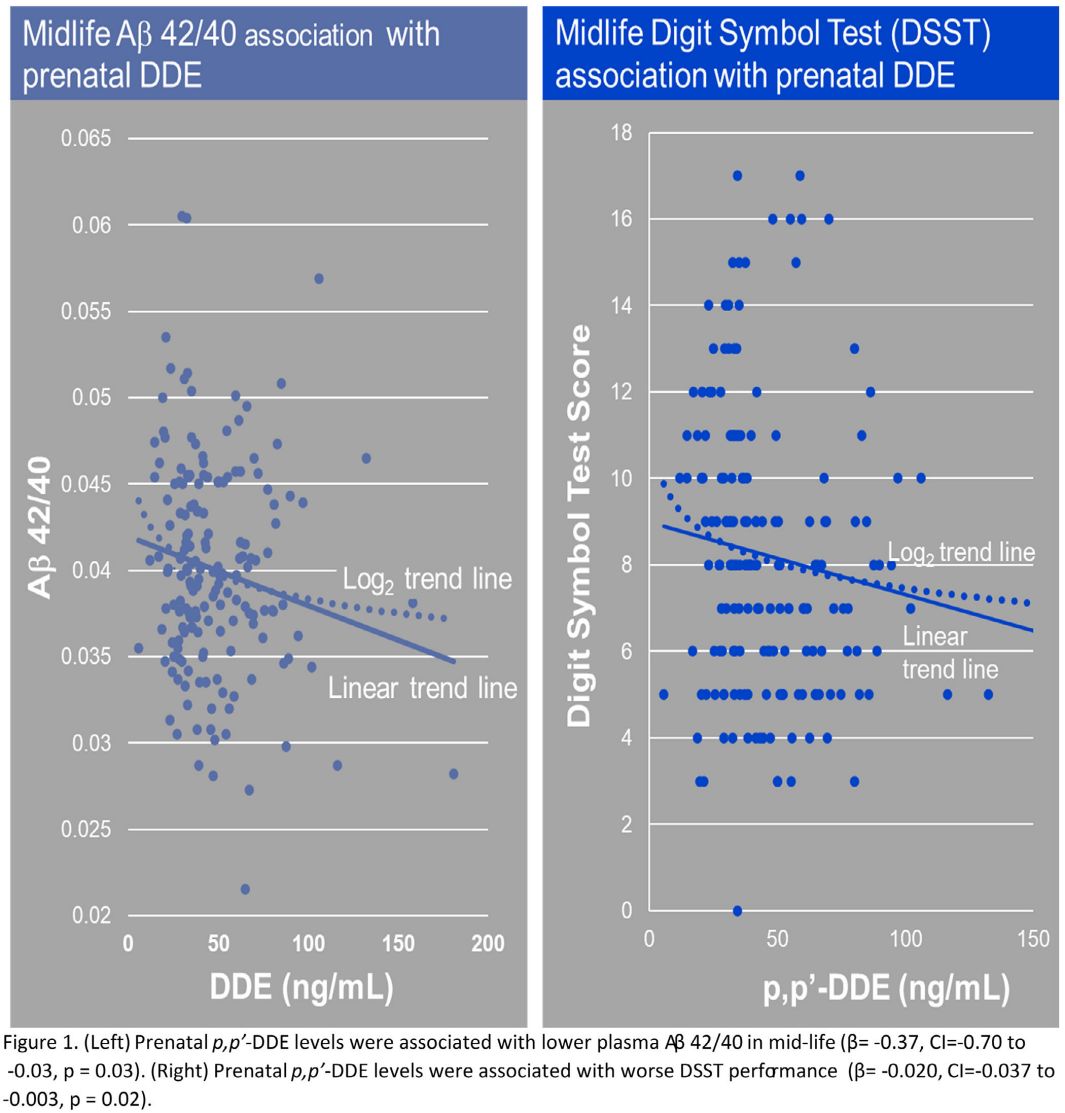# Association of prenatal pesticide exposure with plasma Aβ 42/40 and cognitive function in midlife: evidence from the Child Health and Development Studies

**DOI:** 10.1002/alz70860_107213

**Published:** 2025-12-23

**Authors:** Jason R Richardson, Isha Mhatre‐Winters, Piera Cirillo, Pam Factor‐Litvak, Nickilou Krigbaum, Young‐Mi Go, Dean P Jones, Barbara Cohn

**Affiliations:** ^1^ Isakson Center for Neurological Disease Research, College of Veterinary Medicine, University of Georgia, Athens, GA, USA; ^2^ Child Health and Development Studies, Public Health Institute, Oakland, CA, USA; ^3^ Columbia University Mailman School of Public Health, New York, NY, USA; ^4^ Emory University School of Medicine, Atlanta, GA, USA

## Abstract

**Background:**

The influence of environmental exposures as risk factors for Alzheimer's and Related Dementias (ADRD) is increasingly being recognized. Our prior work found that higher serum concentrations of DDE, the highly persistent metabolite of the pesticide DDT increased risk of Alzheimer's disease and worse cognitive performance. Experiments in animal models revealed that DDT and DDE decrease cognitive function and increase amyloid pathology, which is reflected by lower plasma Aβ 42/40 ratio. However, little is known about the effects of prenatal DDE exposure on plasma Aβ and cognitive function in humans.

**Method:**

Offspring born into The Child Health and Development Studies (CHDS) pregnancy cohort from 1959‐1963 in Oakland, CA were recruited for a diverse follow‐up study of health disparities in 2010. Participants completed cognitive function assessments (Digit Symbol Test; DSST), and provided interview data and blood samples, in their late 40's to early 50's. Aβ 42/40 was measured from the stored plasma taken in midlife using the Quanterix Neurology 3‐Plex A kit. Prenatal *p*,p'‐DDE, measured in archived maternal pregnancy serum, was available for this sample, *n* = 179. Associations were estimated in generalized linear regression models, adjusted for race, sex, educational attainment and APOE genotype.

**Result:**

Prenatal *p*,p‐DDE (log‐transformed) levels were associated with lower plasma Aβ 42/40 in mid‐life (β = ‐0.37, CI=‐0.70 to ‐0.03, *p* = 0.03). For the DSST, prenatal *p*,p'‐DDE levels were associated with worse performance (β = ‐0.020, CI = ‐0.037 to ‐0.003, *p* = 0.02). Associations were robust to multiple alternative modeling approaches. Adjustment for covariates had minimal influence on reported associations.

**Conclusion:**

Prenatal exposure to *p*,p'‐DDE is associated with lower plasma Aβ 42/40 and worse performance on the DSST in midlife. Identification of individuals or populations more highly exposed to DDT/DDE may identify those at increased risk for ADRD later in life and allow for early intervention and prevention from progression to ADRD.